# Application of Nano-Crystalline Diamond in Tribology

**DOI:** 10.3390/ma16072710

**Published:** 2023-03-28

**Authors:** Yue Xia, Yunxiang Lu, Guoyong Yang, Chengke Chen, Xiaojun Hu, Hui Song, Lifen Deng, Yuezhong Wang, Jian Yi, Bo Wang

**Affiliations:** 1 College of Materials Science and Engineering, Zhejiang University of Technology, Hangzhou 310014, China; 2 Key Laboratory of Marine Materials and Related Technologies, Zhejiang Key Laboratory of Marine Materials and Protective Technologies, Ningbo Institute of Materials Technology and Engineering, Chinese Academy of Sciences, Ningbo 315201, China; 3 Chair of Functional Materials, Department of Materials Science & Engineering, Saarland University, 66123 Saarbrücken, Germany

**Keywords:** nano-crystalline diamond, tribology, friction reduction, wear resistance

## Abstract

Nano-crystalline diamond has been extensively researched and applied in the fields of tribology, optics, quantum information and biomedicine. In virtue of its hardness, the highest in natural materials, diamond outperforms the other materials in terms of wear resistance. Compared to traditional single-crystalline and poly-crystalline diamonds, nano-crystalline diamond consists of disordered grains and thus possesses good toughness and self-sharpening. These merits render nano-crystalline diamonds to have great potential in tribology. Moreover, the re-nucleation of nano-crystalline diamond during preparation is beneficial to decreasing surface roughness due to its ultrafine grain size. Nano-crystalline diamond coatings can have a friction coefficient as low as single-crystal diamonds. This article briefly introduces the approaches to preparing nano-crystalline diamond materials and summarizes their applications in the field of tribology. Firstly, nano-crystalline diamond powders can be used as additives in both oil- and water-based lubricants to significantly enhance their anti-wear property. Nano-crystalline diamond coatings can also act as self-lubricating films when they are deposited on different substrates, exhibiting excellent performance in friction reduction and wear resistance. In addition, the research works related to the tribological applications of nano-crystalline diamond composites have also been reviewed in this paper.

## 1. Introduction

Since the preparation of diamond from graphite under high temperature and pressure conditions was reported for the first time by Bundy et al. in 1955 [1], the road to synthetic diamonds has been opened. Though nanoscale diamond particles were first prepared through explosions in the Soviet Union in the 1960s, they remained largely unknown in other parts of the world until the late 1980s. In the 1990s, numerous important breakthroughs led to a broader interest in these nanoparticles [2,3,4,5,6,7,8,9,10,11,12,13,14,15,16,17,18]. These nanoparticles are known as nano-crystalline diamond (NCD) [19]. NCD is a kind of diamond with a grain size below 100 nm. The ultrafine grain size of NCD renders it not only the high mechanical properties [20,21,22,23,24,25], optical properties [26,27,28] and stable chemical properties [29,30,31,32] of diamond itself but also surface and interface effects, small size effects and quantum size effects [33,34,35]. Meanwhile, the biocompatible and non-toxic nature of NCD [36] has attracted a wide range of research and applications in the fields of tribology, optics [37,38,39,40,41,42], quantum information [43,44] and biomedicine [45,46,47,48,49,50,51].

Lubricating is an effective way to improve the efficiency of energy use. In recent years, with the increasing risk of an energy shortage, the properties of anti-wear and friction reduction have become more and more critical for mechanical systems to reduce their energy loss caused by friction. Therefore, research in the field of lubrication has been of great interest. NCD has excellent properties such as small particle size, high hardness, stable chemical properties, good adsorption and they are non-toxic, which means NCD has a wide application in tribology [30,33,34,35,36]. Firstly, NCD particles can be added to fluid lubricants as lubrication additives. During friction, spherical and quasi-spherical NCD particles would be embedded in the contact micro-concavities between the rubbing pairs, giving them good load-bearing capacity on the friction interface. In addition, in the process of sliding friction, firstly, NCD particles will polish the rough contact surfaces. After polishing by NCD particles [52], the rolling of the unembedded NCD particles between the smooth friction surfaces forms a “ball-bearing effect”, leading to the transformation of the friction mode from the original pure sliding friction between the friction pairs into a mixture of sliding and rolling frictions. Moreover, NCD particles will participate in the formation of a tribological film on the friction interfaces through the suction and friction extrusion, which improves the hardness and anti-wear performance of the friction sub-surface. NCD particle additives are used in a wide range of lubricants, polishing fluids and in the lubrication of body fluids in biological joints. NCD films can also be deposited on a substrate, acting as an excellent friction coating to protect the substrate [53,54,55]. NCD films are mostly used for the coating of blade end mills and drills. With the expansion of applications, the NCD coating of artificial joints in biology also offers new ideas to solve the problem of joint wear [56,57,58,59,60]. In the end, NCD powders can be synthesized into composites with other materials by electroplating, chemical plating, sintering, etc. These make NCD play a very important role in lubrication application as an ideal lubricating material. This paper reviews the preparation of NCD in the form of particles, thin films and composites, as well as the current applications of NCD in tribology.

## 2. Synthesis of NCD

The structural shape of NCD largely determines the performance of NCD in tribology and its application fields. Structural features of NCD vary depending on different synthesis methods. The main synthesis approaches for NCD include detonation, high temperature and high pressure (HTHP), as well as chemical vapor deposition (CVD) [61,62,63,64,65,66]. The HTHP method is to prepare diamonds through simulating the growth environment of natural diamonds by the cubic press, in which graphite is mainly used as the carbon source. Since diamond is a thermodynamically stable phase at high pressure, the thermodynamic entropy for the conversion of graphite to diamond is consistent at high pressure. However, the reaction activation energy for the conversion of graphite to diamond is high, so the catalyst should be employed to reduce the activation energy during the growth process. The detailed growth process is to put the carbon source (for example, graphite) and the seed crystalline diamond into the high- and low-temperature zones, respectively, making the temperature of the carbon source higher than that of the seed crystal. Driven by the temperature gradient, carbon will diffuse to the seed crystalline diamond and thus crystallize and precipitate on the seed crystal to obtain the diamond.

The detonation synthesis method is to prepare NCD powders by shock wave treatment of graphite-metal mixtures or by the explosive impact of organic compounds with high carbon content and relatively low oxygen content. The thermodynamically favorable conditions for the formation of graphene layers in decomposition products of explosives and their subsequent consolidation with the formation of monocrystalline graphite particles are implemented at the first stage. The transformation of the latter into a diamond is performed in accordance with the martensite mechanism [67]. The NCD powders prepared by the detonation synthesis have a small size and are spherical or oval in shape. As a kind of lubricating additive, it tends to roll in the friction surface to form the “ball-bearing effect”, thus reducing the coefficient of friction (COF) and wear. This is an important reason why NCD prepared by detonation synthesis is generally used as a lubricant additive.

Chemical vapor deposition can prepare a desired thin film material on a substrate using a gas phase precursor that reacts chemically under specific deposition conditions. A mixture of carbon-containing gases (for example, methane) and hydrogen is decomposed at high temperatures and low pressures, below standard atmospheric pressure, forming plasma containing hydrocarbon radicals. Diamond is deposited on the substrate through the adsorption and reaction of the hydrocarbon radicals. The stable phase of carbon is the graphite phase at atmospheric pressure. Therefore, a continuous supply of atomic hydrogen in plasma is required to decompose other products surrounding the sp^3^ structure during the growth of diamond by CVD. The advantages of NCD films prepared using the CVD process over other synthesis techniques include (1) low pressure and low temperature, (2) prevention of non-carbon impurities and (3) ease of doping the diamond phase with other elements (e.g., boron, nitrogen, phosphorus) to modify its properties [68]. NCD films prepared by the CVD method are mainly deposited on the surfaces of friction pairs. By taking advantage of its low COF, ultrafine grains, low roughness and high hardness, the friction pairs coated by NCD film have excellent tribological performances.

## 3. NCD for Tribological Applications

### 3.1. NCD Lubricant Additives

**Figure 1:**
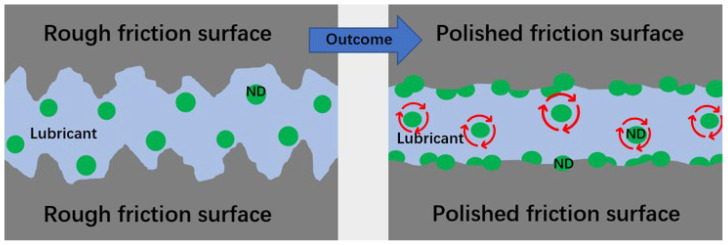
The polishing effect of NCD particles in lubricant and ball-bearing effect of NCD powders during friction process.

In virtue of their small particle size, high hardness and chemical stability, NCD powders are considered kinds of promising materials in the field of lubricant additives. The underlying mechanism of the NCD particles acting as a lubrication additive lies in their abilities to polish the friction surface and produce the “ball-bearing effect” at the interface, as well as the tribological film formed by the NCD particles embedded onto friction surfaces, thus achieving an excellent performance of anti-wear and friction reduction. Figure 1 illustrates the polishing effect of the lubricant containing NCD powders on the friction surfaces, as well as the “ball-bearing effect “generated during rubbing. Tribological performance of NCD lubricant additives is summarized in Table 1.

**Figure 2:**
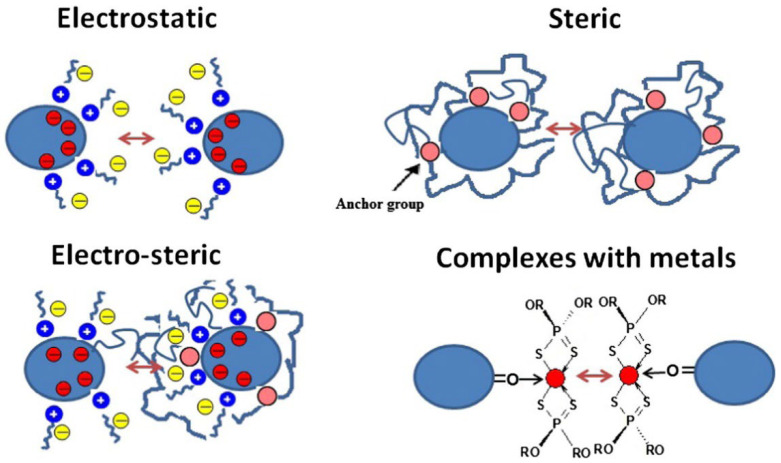
Four stabilizing mechanisms for nanoparticles [69]. Adapted with permission.

To be effectively used as a lubricant additive, stable dispersion ability is necessary for NCD particles. For nanoparticles, there are four stabilizing mechanisms: (i) electrostatic stabilization, (ii) steric stabilization, (iii) electro-steric stabilization and (iv) complexes with metals stabilization. As shown in Figure 2 [69], the electrostatic stabilization is governed by ionogenic groups on the surface or adsorbed ions; the steric stabilization is provided by the presence of branched groups; the electro-steric stabilization is the combined effect of the electrostatic stabilization and the steric stabilization and the complexes with metals stabilization are the stabilization with a coordination compounds [69]. These four stabilizing mechanisms are also suitable for NCD powders as lubricant additives. According to the above four stabilizing mechanisms, it can be better to solve the problem of stable dispersion with NCD particles.

Compared to traditional oil-based lubrication additives, such as metal nanoparticles (Cu, Ni) and compound nanoparticles [SiO_2_, Mg_3_(BO_3_)_2_], NCD powders have much smaller size and higher hardness, resulting in a great improvement in the wear resistance [70]. Xu et al. [52] and Novak et al. [71] employed the explosively synthesized NCD powders as additives to paraffin oil. The tribological properties of the two-phase lubricant of paraffin oil and NCD particles were investigated with a friction and wear tester in a relative humidity (RH) of 40–50% at ambient temperature. The results showed that, due to the high hardness of NCD powders, friction surfaces could be polished during the friction process, while NCD powders will produce the “ball-bearing effect” at the interface, rendering the two-phase lubricant to possess excellent load-bearing capacity, anti-wear and friction-reducing properties.

**Figure 3:**
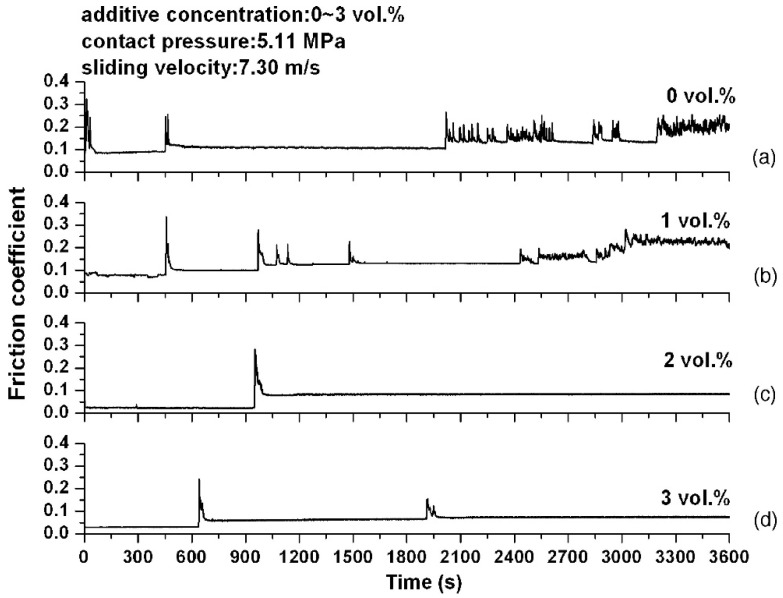
Variation of friction coefficient vs. time under the contact pressure of 5.11 MPa with a sliding velocity of 7.30 m/s. The NCD powder concentrations are (**a**) 0 vol.%, (**b**) 1 vol.%, (**c**) 2 vol.% and (**d**) 3 vol.% [72]. Adapted with permission.

Chu et al. [72] investigated the anti-scuffing properties of NCD particles as oil additives. The lubricating oil, which contains NCD powders with various volumetric concentrations, was used to evaluate their tribological performances and scuffing resistance in the block-on-ring adapter operating at different speeds and load conditions. The results showed that the addition of NCD particles could improve the anti-wear performance by reducing the number of surface failures caused by scuffing. Figure 3 shows the variation of the friction coefficient with test time for the four NCD additive concentrations (0 vol.%, 1 vol.%, 2 vol.% and 3 vol.%). It exhibited a significant reduction of friction at a low NCD concentration of 2% or 3% [73]. To fully give play on its reductions of friction and wear, NCD powders also meet the problem of aggregation in oil. In order to improve the dispersity of NCD powders in anti-wear hydraulic oil (AHO), Li et al. [58] prepared acidified and heat-treated NCD and then mixed it with a variety of additives, such as oleyl amine, polyisobutylene succinimide (T154) and high alkaline calcium sulfonate (T106). After acidification and heat-treatment, the average size of the NCD powders decreased. The existence of other additives could increase the oil solubility of NCD powders and thus prevent their aggregation, giving rise to the enhancement of the dispersion stability of NCD powders in AHO.

Transportation vehicles account for about 19% of the world’s energy consumption and 23% of the total greenhouse emissions every year. Wear and friction of the mechanical elements cause energy dissipation in vehicle engines. The engine-generated power is diminished in the range of 17–19% due to frictional losses. [74] High friction between the mechanical components in a vehicle poses a significant challenge to its efficiency of energy use and thus increases the emission of greenhouse gas. Therefore, the friction reduction achieved through employing engine lubricating oils keeps and attracts intensive attention as an effective way to improve engine efficiency [69,75]. Huang et al. [76] investigated the effect of NCD powders in lubricating oil on the tribological performances between CrN-coated piston rings and chromium-plated and BP-alloyed iron cylinder liners by using the reciprocating sliding tribometer, which is one of the key friction pairs in the internal combustion engine.

Apart from acting as lubricating additives individually, NCD powders have been researched in the preparation of hybrid lubricating additives. As a mixing additive, Ivanov et al. [69,77] reported the tribological performance of stable colloidal dispersions consisting of detonation NCD powders and other lubricant additives in mineral oil, greases as well as polyalphaolefin (PAO) oil. The results demonstrated that the friction and wear were noticeably reduced after adding the detonation NCD powders into the greases and oils. Moreover, the size of the aggregated NCD powders played an important role in the lubricating properties of the compositions. Except for the enhancement of the friction reduction performance, the combination of NCD powders and poly tetra fluoroethylene (PTFE) particles in greases and oils will also improve the extreme pressure failure load. This is derived from the formation of a stable polymer-based friction film under the synergy between NCD powders and PTFE particles. The formed friction film can withstand multiple deformation modes without failure. Raina et al. [78] examined the influence of NCD particles as secondary additives on the oil lubrication of steel-aluminum friction (hard-soft contact). It is found that NCD particles acting as secondary additives under a low concentration of 0.2 wt.% can improve the oil lubrication performance of hard-soft contacts in engineering systems.

In addition, to being employed as a lubricating additive in oil-based lubricants, NCD powders have exhibited their advantages as an additive in a water-based lubricants. Both researches conducted by Wang et al. [79] and Alias et al. [80] testified that the tribological properties were improved after adding 0.001 wt.% NCD in water, which was investigated by utilizing a ball-on-disk tribometer. The tribological performance of NCD additives in water has also been investigated by experiments and molecular dynamic simulations (MD) by Mirzaamiri et al. [81]. The results showed that the introduction of NCD powders in water significantly improved friction reduction and wear resistance by 70% and 88%, respectively. They stated the improved tribological performance is mainly attributed to the ball effect of NCD powders and verified the rolling motion of NCD particles by MD simulations. Besides, it is also found that ultrasonication treatment can further improve the lubricating properties of NCD during the preparation of nano-lubricants.

In virtue of their excellent biocompatibility, NCD powders are also considered a kind of promising joint lubrication additive in biomedical human bone joint friction. Shirani et al. [82] demonstrated that the COF and wear of the Ti artificial joints were reduced by three folds and two orders of magnitude, respectively, through injecting simulated body fluids containing NCD powders with a concentration of less than 0.2 wt.%. In another work, Shirani et al. [83] also suggested that NCD powders have a great potential for treating artificial joint complications by intra-articular injection, benefiting from their excellent biocompatibility combined with outstanding anti-wear and antibacterial properties. Very recently, Chen et al. developed a diamond nanosheet coating on Ti alloy through self-assembly, achieving reductions of 54% and 98% for COF and wear, respectively, with the synergistic effect of silk fibroin.

**Table 1:** Summary of tribological performance for NCD lubricant additives.

Lubricating System	Tribological Test and Performance					Refs.
	Method	Environment	Counterpart	COF (Original)	Wear (Original)	
1% NCD/ paraffin oil	ball-on-disc	ambient conditions, 50–200 N, 50 Hz, 0.2 m/s	GCr15 steel; #45 steel	0.08 (0.18–0.13)	0.2 × 10^−3^ mm^3^ (1 × 10^−3^ mm ^3^)	[52]
3 vol.% NCD/ CPC R68 oil	block-on-ring	3.54–5.11 MPa, 4.87–7.30 m/s	SKD11 steel, SKD61 steel	0.071 (0.089)	0.0206 g (0.0367 g)	[72]
0.03–0.05% NCD/ 0.3% PAO	ring-on-ring; four-ball; block-on-ring	314 N, 500–1500 rpm; 196 N, 1460 rpm; 300 N, 200 rpm	52100 steel; 5140 steel; SAE 01 steel, stainless steel	0.043–0.001 (0.195–0.052)	0.350 mm (0.646 mm)	[73]
1% NCD/ HVI-5 oil	reciprocating sliding	150 °C, 200 rpm, 10–40 MPa	CrN, Cr/BP	0.195–0.215 (0.255–0.27)	1.5 μm (4 μm)	[76]
0.001 wt.% NCD/ water	ball-on-disk	ambient conditions, 5–15 N, 10–100 mm/s	SiC	0.01 (0.15–0.19)	1.5 × 10^−6^ mm^3^/N·m (3 × 10^−6^ mm^3^/N·m)	[79]
0.01 wt.% NCD/ water	ball-on-disk	1.88 N, 40 m/s	SUS304 teel, WC	0.1 (0.4)	<(original)	[80]
0.05 wt.% NCD/ simulated body fluid	pin-on-disk	37 °C, 1 Hz, 0.25 –1 N	Ti	0.2–0.35 (0.4–0.7)	<(original)	[82]
0.45 g/L NCD/ body fluids	ball-on-disk	37 °C, 1 Hz, 0.25–2 N	UHMWPE, Ti	0.15–0.2 (0.2–0.3)	<(original)	[83]

Note: For all tables in this paper, the data in the brackets represent the value without NCD lubricating.

### 3.2. NCD Lubricating Films

**Figure 4:**
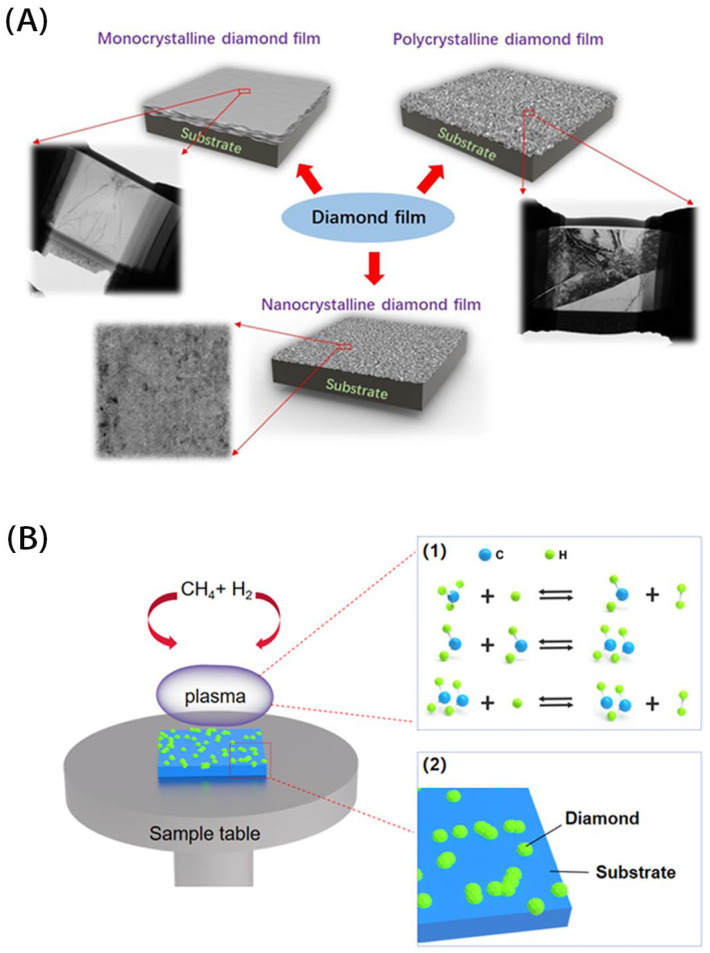
Schematic diagrams of (**A**) different diamond films and (**B**) diamond preparation using CVD ((1) Chemical reactions in plasma, (2) Diamond deposition on the substrate.).

Except for lubricating additives, NCD film can be used as a solid lubricant after depositing it on a substrate using the chemical vapor deposition (CVD) method. Compared to monocrystalline and poly-crystalline diamond films, the grain size for NCD films is below 100 nm, resulting in a smooth surface without polishing, as shown in the schematic diagram in Figure 4A. Besides, NCD films generally contain non-diamond phases in them, for example, the graphite phase, benefiting from the formation of the tribo-layer on the sliding surface and resulting in a low COF. Combined with their high hardness, high wear resistance and good chemical stability, NCD films are considered excellent surface anti-wear modified films. Table 2 summarizes the tribological performance for NCD lubricating films. At present, there are three kinds of CVD equipment for preparing NCD films, namely hot filament CVD (HFCVD) [84,85,86,87,88,89,90], microwave plasma CVD (MPCVD) [91,92,93,94,95,96] and plasma enhanced CVD (PECVD) [97,98,99,100,101]. The schematic diagram of diamond film preparation is shown in Figure 4. Generally, H_2_ and CH_4_ are mixed in a certain ratio and passed into the CVD reaction chamber. The mixed gas is dissociated in the CVD chamber to produce free radical products, which are absorbed onto the substrate and then form the sp^3^ phase of diamond through chemical reactions.

A monolayer NCD film was synthesized by Yoshikawa et al. [102] on a 4-inch silicon wafer using an MPCVD system to investigate its tribological properties for the first time. The COF of the NCD films rubbing against a SiC ball under 0.5 N in air leveled off to a very low value of 0.02 from the large initial value, indicating that NCD films have great potential for tribological applications.

Subsequently, researchers began to deposit NCD films onto other substrates and explore their tribological performance. This further expanded the range of applications in tribology for NCD films. NCD films were prepared by Kumar et al. [103] on three types of steel alloys, including R6M5, 12X2H4A and 38X2MUL, using a linear antenna microwave plasma enhanced chemical vapor deposition (LA-MWPECVD) system [104]. Tribological performances of the deposited NCD films were measured under different conditions. The low COF of the NCD films deposited on steel alloys was observed under a high vacuum at high temperature (HVHT). Moreover, the high abrasion resistance of the NCD films was observed under a high vacuum at room temperature (HVRT). However, the NCD films showed high friction and low wear resistance in the ambient atmosphere at room temperature (AART). The difference in tribological performance is related to the formation of metal oxide (Fe_2_O_3_ and Fe_3_O_4_) layers in the sliding interface. Under HVHT, the oxidation of the NCD film is limited, contributing to achieving a low COF. This research has provided new ideas for wear-resistant coatings on steel alloys. At the same time, NCD film was successfully synthesized by Das et al. [105] on an untreated glass substrate through spontaneous growth at low temperatures (~300 °C), providing a new method for the preparation of NCD films for nano-tribological application. During the deposition, CO_2_ was used as a supplemental gas phase in the (CH_4_ + H_2_) plasma. Furthermore, a specific shade shield was employed to generate a diffusive plasma environment above the growth zone.

Except for the intrinsic material properties, the morphology and microstructure also play a critical role in the tribological performance of NCD films. Peng et al. [106] prepared NCD films with good adhesion onto carbide cement through tuning the Ar/H_2_ ratio by HFCVD. Prior to the preparation of the NCD films, pre-treatments, including sand-blasting and two-step etching, were performed on the carbide cement. It was found that the addition of Ar could enhance grain refinement and tune the morphology of the NCD films. With the addition of Ar, both the COF and wear rate decreased monotonically. The wear rate of the coating prepared with 90% Ar was less than that of the coating prepared without Ar, resulting from the transformation of the wear mechanism from exfoliation being limited by the stress-induced tribo-layer of the sp^2^ phase. Besides, the wear behavior of B-doped NCD films was first investigated by Buijnsters et al. [107]. After doping with a high content of B element, both the hardness and elastic modulus of the NCD films were reduced, which induced an increase in the wear rate. In addition, due to its higher density of grain boundaries, the nucleated surface suffered more severe wear compared to the grown surface. Herein, it can be seen that the doping or the addition of other gases to the working gas during the growth process has an influence on the structure of the NCD films, which directly affects the tribological properties of NCD. Service conditions also are critical factors for the tribological behavior of NCD films. For some friction parts, performing in a water-based environment is unavoidable. Their frictional wear properties may be significantly altered in an aqueous environment. Yan et al. [108] investigated the effect of surface texture at the nanoscale on the frictional wear performance of NCD films under water-based lubrication conditions. The untreated NCD films showed a stable friction state with an average COF of 0.26. Nonetheless, the texturized NCD films showed a beneficial effect on the rapid reduction of the COF, which dropped to a stable value of 0.1. In addition, the wear rate on the counter ball of the texturized NCD films decreased from 4.16 × 10^−3^ to 1.15 × 10^−3^ mm^3^/N∙m, compared to that of the untreated NCD films. The decreased wear rate can be attributed to the achievement of a fluid lubrication state, resulting from the formation of the hydrodynamic fluid film consisting of water and debris. Meanwhile, surface weaving can greatly improve the hydrophilicity of the NCD films. The water contact angle of the NCD films decreased from 94.75° to 78.5° after surface weaving. Their research demonstrated that the NCD films with proper texturing exhibited excellent tribological properties, such as short run-in period, low COF and low wear rate.

**Figure 5:**
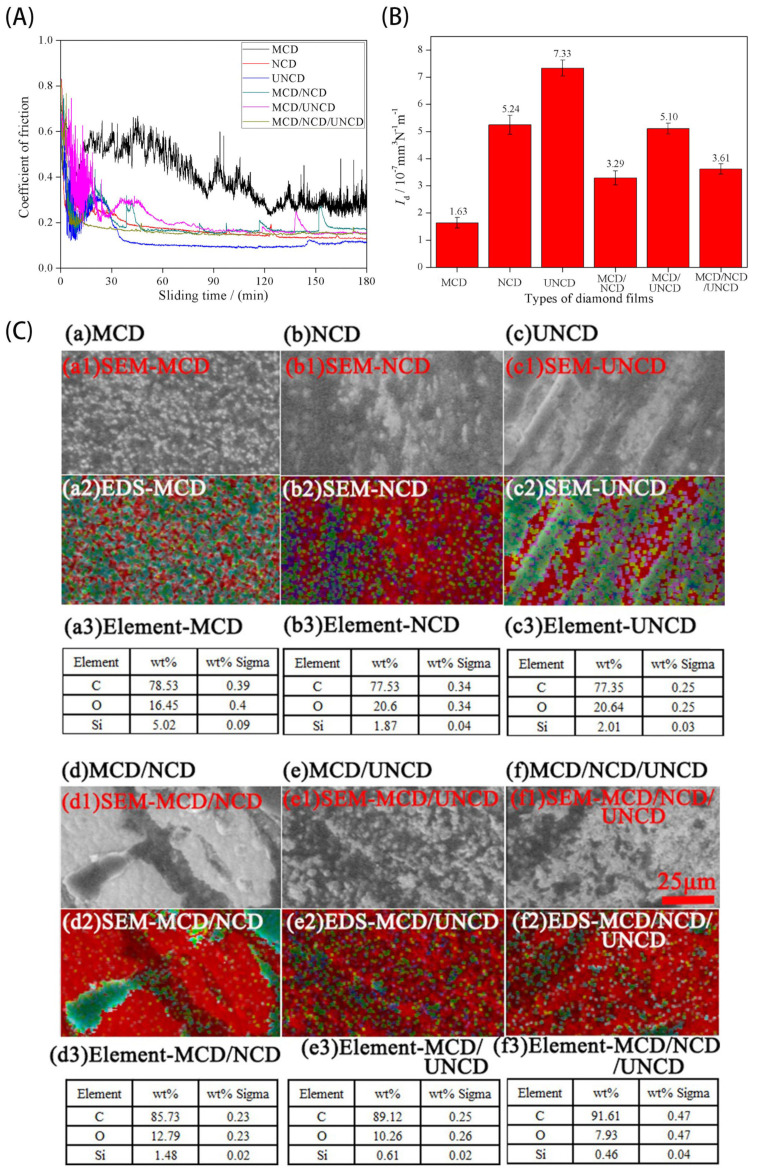
(**A**) Curves of COFs and (**B**) average wear rates of different diamond films against SiC balls. (**C**) Comparisons of SEM images(a1,b1,c1,d1,e1,f1) and EDS mapping(a1,a2; b1,b2; c1,c2; d1,d2; e1,e2; f1,f2) of the transfer film formed on the frictional zones for different polycrystalline diamond coatings of (a) MCD; (b) NCD; (c) UNCD; (d) MCD/NCD;(e) MCD/UNCD; (f) MCD/NCD/UNCD [112]. Adapted with permission.

The grain size of the NCD film also poses a significant influence on its tribological behavior. The value of the COF is related to the surface roughness of the coating. Due to the high crystallinity, monolayer micro-crystalline diamond (MCD) coating strongly bonds with the substrate, leading to enhanced wear resistance and a high COF. In contrast, the monolayer ultrafine nano-crystalline diamond (UNCD) exhibits a low COF and roughness. The performance of the monolayer NCD films falls somewhere between MCD and UNCD [53,109,110,111]. Wang et al. [112] prepared various diamond films (used for fabrication of diamond end mills) using the HFCVD technique on cobalt sintered tungsten carbide (WC-Co6%) substrates, including single-layer MCD, single-layer NCD, single-layer UNCD, double-layer MCD/NCD, double-layer MCD/UNCD and multilayer MCD/NCD/UNCD. In these developed diamond materials, an NCD or UNCD film is fabricated onto the cutting edge, exhibiting excellent chip evacuation capabilities, wear resistance and extending the mill’s life during high-speed machining. Surface morphologies, COF curves and wear rates of different diamond coatings, including the error bars, are shown in Figure 5. Figure 5A,B clearly show the wear amount and the variation curve of the COF for different coatings. The MCD film and multilayer film containing MCD film exhibit high wear resistance, resulting from the higher hardness of MCD films. Especially for the MCD film, no wear track can be found in the SEM image after testing (Figure 5A). However, from Figure 5A, it can be seen that the MCD film and multilayer film containing MCD film suffer from high friction. In the SEM images and corresponding EDS analysis, it can be concluded that tribo-film is difficult to form on the MCD film, resulting in its high COF value. For multilayer films consisting of MCD and NCD film or/and UNCD film, in which the MCD film acts as the base layer, tribo-films can be produced on them during rubbing, as shown in Figure 5C(d–f). Therefore, multilayer films consisting of MCD and NCD film or/and UNCD film can possess the combined abilities of anti-friction and wear resistance.

In addition to cutting tools, a high-load mechanical seal ring is another noteworthy application for multilayer diamond coating [113]. A mechanical seal friction system based on multilayer MCD/NCD coating was proposed by Shabani et al. [113]. The multilayer MCD/NCD coating was grown on a Si_3_N_4_ sealing ring using HFCVD. Their study demonstrated that complete sealing conditions were achieved in the limiting pv (product of the pressure of the medium being sealed “p (Pa)” and the average circumferential velocity of the end face “v(m/s)”) value range of 0.75–5.5 MPa/ms in pressurized water. The coated diamond films can prevent the adhesion between friction pairs made by polar materials (Si_3_N_4_, SiC, etc.) under high load.

Intriguingly, due to its low COF, high wear resistance and excellent biocompatibility, NCD has also been applied as a solid lubricant for artificial implants. Metal implants used in hip and knee joints tend to fail, induced by the combined effects of wear and corrosion. NCD coatings on metal implants may be potential candidates to overcome this problem. Gopal et al. [114] investigated the tribo-corrosion and electrochemical behavior of NCD coatings deposited on Ti alloys, such as Cp-Ti, Ti-6Al-4V and Ti-13Nb-13Zr, using the HFCVD technique. However, the application of NCD in implants still meets some drawbacks, e.g., the coating blistering under wear and corrosion.

**Table 2:** Summary of tribological performance for NCD lubricating films.

Lubricating System	Tribological Test and Performance					Refs.
	Method	Environment	Counterpart	COF	Wear	
MPCVD NCD on silicon	ball-on-disk	ambient conditions, 0.5 N, 10 cm/s	SiC	0.02	\	[102]
LA-MWPECVD NCD on steel alloys	pin-on-disk	AART/HVRT/HVH, 1 N, 3 cm/s	100 Cr6	0.38–0.42	2 × 10^−6^ mm^3^/N·m	[103]
HFCVD NCD on carbide cement	ball-on-disk	ambient conditions, 4/20 N, 5 Hz, 0.03 m/s	Si_3_N_4_	0.1	4 × 10^−9^ mm^3^/N·m	[106]
HFCVD B-doping NCD on silicon	ball-on-disc	ambient conditions, 2 N, 5 Hz	Al_2_O_3_, diamond-coated ball	\	1.5 × 10^−7^ mm^3^/N·m	[107]
HFCVD NCD on WC-Co 6%	ball-on-disc	ambient conditions, 20 N, 0.168 m/s	WC–Co	0.1	1.15 × 10^−3^ mm^3^/N·m	[108]
HFCVD NCD on WC-Co substrate	ball-on-disc	ambient conditions, 4 N, 400 r/min	SiC	0.137	5.24 × 10^−7^ mm^3^/N·m	[112]
HFCVD MCD/NCD on WC-Co substrate				0.173	3.29 × 10^−7^ mm^3^/N·m	[112]
HFCVD MCD/NCD/UNCD on WC-Co substrate				0.143	3.61 × 10^−7^ mm^3^/N·m	[112]
HFCVD NCD on Ti alloy	pin-on-disc	28 N, 1 Hz, 20 mm/s	UHMWPE	\	\	[114]

### 3.3. NCD Lubricating Composites

**Figure 6:**
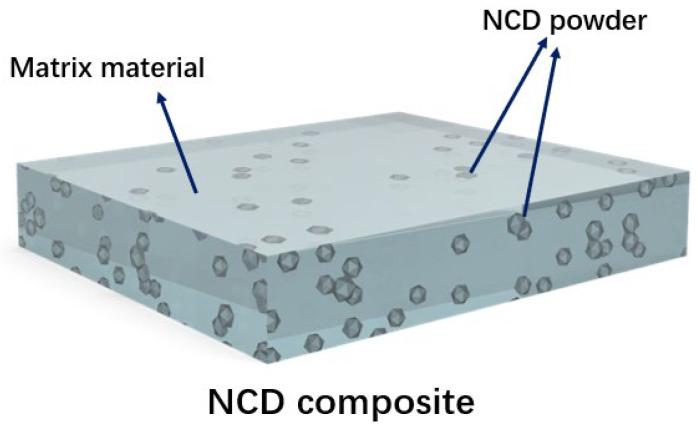
Illustration of the structure of NCD composite materials.

In recent years, due to its outstanding properties of high hardness, small size, chemical stability and high thermal conductivity, NCD has attracted much attention in the study of blending NCD powders with other materials to prepare composites with high tribological performance, as shown in Figure 6. Its unique properties make NCD powder a promising candidate as a reinforcing material [115]. NCD composites are new materials prepared by adding NCD powders into other materials (for example, metal matrix composites such as Ni [116,117,118,119], Al [120,121,122,123], Fe [124], Mg [125,126], Ti [127,128,129,130], Cu [131,132,133], etc.) through electroplating, chemical plating and hybrid sintering. Since Ni and Al have aesthetics, excellent performance and practicality, they are widely used as protective coatings in a wide range of applications [116,117,118,119,120,121,122,123,124]. The hardness and wear resistance of the NCD-Ni or NCD-Al composite coatings will be enhanced after introducing the ultrahard phase of NCD powders. In the same way, the addition of NCD powders to Fe, Ti or Cu coating also leads to new NCD composites with excellent frictional properties. For example, Ti-NCD composite coating can be applied on artificial implants of hip and knee prostheses. Table 3 is an overview of the tribological performance of NCD composite coatings and NCD composite bulk materials. This is the summary of tribological performance for NCD lubricating composites, as shown in Table 3.

#### 3.3.1. Ni-Based NCD Composites

Nickel plating has a wide range of applications as a protective coating, benefiting from its excellent corrosion resistance, high hardness and abrasion resistance. In recent years, the development of composite plating has made it possible to deposit nickel composite coating containing hard particles, whose hardness and wear resistance are higher than that of pure nickel plating.

As NCD particles have their own advantages of high hardness and good abrasion resistance, much research has been carried out on the development of composites consisting of Ni and NCD particles. Composite plating is considered a feasible and economical technique for the preparation of such kinds of composites. In order to investigate the effect of the crystal size of nickel on the tribological properties of Ni-NCD composites, Wang et al. [116] prepared composite coatings consisting of NCD particles embedded in nano-crystalline nickel (n-Ni) and micro-crystalline nickel (m-Ni) matrix materials using conventional electrodeposition methods. It was shown that, for m-Ni coating systems, the incorporation of NCD particles changed the crystal growth from (119) orientation to random orientation and produced a higher-density structure with smaller grain sizes. In the case of n-Ni coating, the intercalation of NCD powders in the n-Ni matrix had no effect on the crystal weaving and microstructure of the matrix. Under dry sliding conditions, the wear rate of m-Ni/NCD composite coating is about half of that of the pure m-Ni coating. The wear rate of n-Ni coatings is approximately reduced by one order of magnitude from that of pure m-Ni coatings. Surprisingly, the n-Ni/NCD composite coating showed poor wear resistance compared to pure n-Ni coating.

As the surface of nickel-phosphorus plating (Ni-P) is amorphous, on the one hand, the corrosion resistance is particularly excellent. On the other hand, it is also shown to be in a fundamentally flat state with self-wetting properties, resulting in a low COF and high abrasion resistance. In order to obtain composites with improved tribological properties, researchers devoted themselves to the development of Ni-P/NCD composites. Xu et al. [115] successfully doped the NCD powders into a Ni-P matrix by chemical plating. The results showed that NCD powders could significantly improve the mechanical and tribological properties, as well as the corrosion resistance of the composite coatings. The prominent effect of NCD on the tribological properties of Ni-P/NCD composite coatings can be attributed to the synergic effect of the near-spherical shape and the super hardness of NCD powders. In addition, the Ni-P/NCD coating has better corrosion resistance than the Ni-P coating. The excellent anti-corrosion properties of the Ni-P/NCD composite coating derive from the favorable chemical stability and small size of the NCD powders, which reduce the size of the pores in the composite coating and prevent the spread of corrosive pits. Karaguiozova et al. [117] also conducted research on Ni-NCD and Ni-P/NCD composites. Besides that, they also developed a technique for improving the tribological and mechanical properties of iron alloys, including steel 17CrNiMo6 and spheroidal graphite cast irons, on the basis of chemical nickel plating. A new nanocomposite coating of Ni-P/NCD was obtained by the sol-gel method without electrodeposition. NCD solution was added directly into the chemically plated Ni-P solution. In order to facilitate their uniform distribution in the coating, a surfactant was also added to obtain well-dispersed NCD powders in the electroless solution. The results showed enhanced hardness and wear resistance of Ni-P/NCD compared to coatings without NCD particles.

As described above, the addition of NCD has led to a significant improvement in the frictional properties of Ni-based composites. Other research related to Ni/NCD composites, such as the suitability of the coating, the requirements of the preparation conditions and new preparation methods, are all underway. Liu et al. [118] prepared Ni-P/NCD composite coatings with good tribological properties without adding any surfactant and investigated the wear mechanism of the coatings. It was shown that the dispersion property of NCD powders was significantly improved in the plating solution after coating a layer of alumina on the surface. The NCD powders with a core-shell structure were uniformly deposited in the plating layer to play the “micro-roller ball” effect, which reduced the COF of the plating layer and enhanced its wear resistance simultaneously. Ekoi et al. proposed a new approach to prepare Ni/NCD nanocomposite coating with high wear resistance [119]. The preparation of the Ni/NCD nanocomposite coating involves the spray deposition of a suspension containing Ni and NCD nanoparticles onto the stainless steel substrate using an atomizer, followed by sintering in a microwave plasma system. The as-prepared Ni/NCD nanocomposite coatings are uniform and have good adhesion. It has been shown that the stability of the Ni/NCD nanoparticle suspension depends on both the employed solvent and the concentration of NCD particles in the suspension. At high NCD powder concentrations, the suspension can remain stable for several months. The morphology of the sintered coating is “fibrous” at lower temperatures and gradually changes into a granular structure as the sintering temperature increases. The roughness and thickness of the coating usually decrease with the increasing sintering temperature. When increasing the NCD powder concentration to above 15%, the roughness and thickness also became correspondingly larger. The addition of NCD powders into Ni significantly improved its wear performance by 126 times. In addition, the results showed that the wear rate is mainly influenced by the concentration of NCD powders.

#### 3.3.2. Al-Based NCD Composites

Due to its poor performance in hardness and wear resistance, the application in tribology is limited for Al plating. However, a composite coating of Al with NCD powder will improve its hardness, making its tribological application become possible [120,121]. Woo et al. [122] successfully prepared dense Al/NCD composite coatings by low-pressure cold spray deposition of the ball-milled powders containing NCD with a concentration of 10 wt.%. It was found that cold spray deposition produced a dense Al/NCD composite coating with enhanced hardness and elastic modulus compared to the raw materials [122].

**Figure 7:**
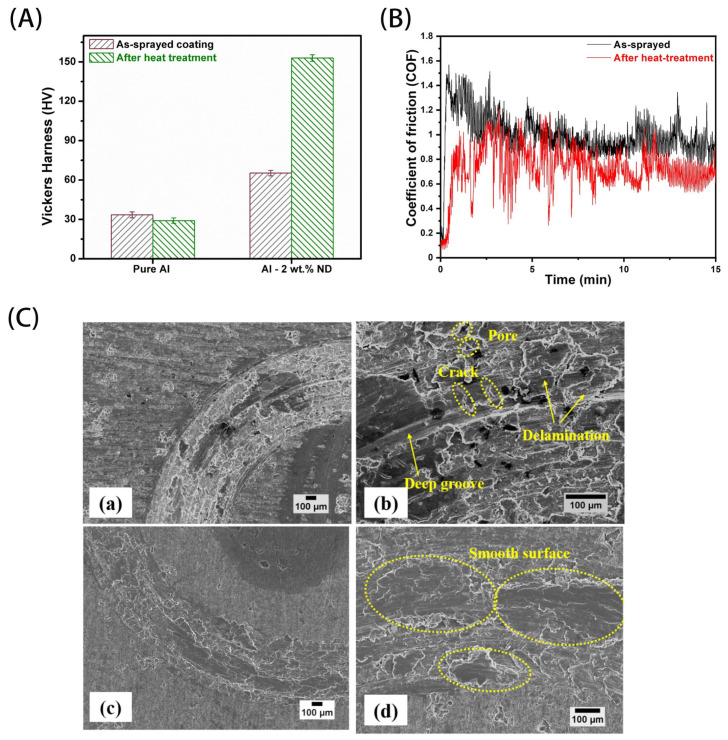
(**A**) Microhardness and (**B**) COF variation of cold-sprayed pure Al and Al-2 wt.% ND coating before and after heat treatment. (**C**) SEM images of wear tracks of (a,b) as-sprayed and (c,d) heat treated Al-2 wt% ND coating [123]. Adapted with permission.

Loganathan et al. investigated the effect of heat treatment on the tribological properties of a uniformly diffusely distributed Al/NCD composite coating prepared by the cold spray technique, as shown in Figure 7 [123]. As shown in Figure 7A,B, both microhardness and anti-friction performance were improved after heat treatment for the Al/NCD composite. The improved anti-friction performance was induced by the high hardness, density and toughness of the coating, as well as the graphitization of NCD powders after heat treatment. To understand the wear mechanism of different composites, wear tracks were investigated using SEM observations. From Figure 7C, it can be seen that the track on the composite after heat treatment has a smooth surface (Figure 7C(b)), without cracks, pores, deep grooves or delamination on the track of composite prior to heat treatment (Figure 7C(d)). This demonstrates the great potential of heat-treated Al/NCD composites as wear-resistant coatings [123].

#### 3.3.3. Fe-Based NCD Composites

**Figure 8:**
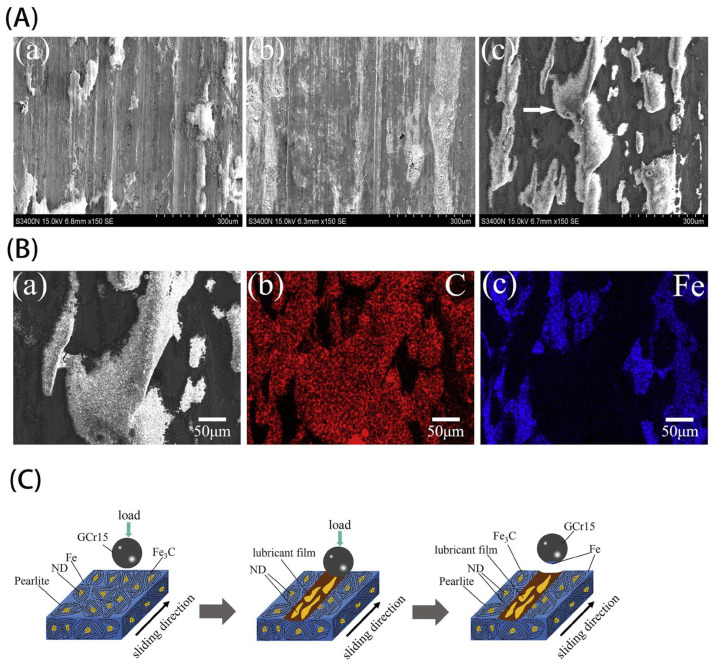
(**A**) SEM images of the wear scar in the sintered Fe/NCD composites: (a) 0, (b) 1 and (c) 2 wt%. (**B**) SEM image(a) and EDX spectra for element of (b) C, and (c) Fe on the wear scar in the Fe/2 wt% ND composite. (**C**) Schematic diagram showing the wear mechanism in the Fe/2 wt% ND composite [124]. Adapted with permission.

(Fe/NCD) were also verified to have outstanding tribological properties. Huang et al. [124] prepared Fe-based composites reinforced by NCD powders through hot-press sintering. The effects of the NCD concentration on the mechanical and tribological properties of the sintered Fe/NCD composites were investigated, as shown in Figure 8. Due to the reaction between Fe and NCD during the sintering process, a pearlite microstructure was formed. The presence of NCD provides higher hardness, compressive and flexural strengths. Compared to the sintered iron, the hardness, compressive and flexural strengths of the composites containing 1 wt.% NCD were increased by 51.5%, 37.4% and 76.2%, respectively. In addition, the COF and wear resistance of the Fe/NCD composite increased with the increasing NCD concentration. In Figure 8A, the worn surface of Fe/1 wt% NCD composite is smoother, and the sliding grooves are shallower compared to that of sintered Fe, indicating that the wear mode changed from adhesive wear to a mixture of adhesive and abrasive wear after introducing NCD powders. However, the stripe-shaped films with high carbon content were formed on the worn surface of the Fe/2 wt% NCD composite (Figure 8B), caused by the unreacted NCD particles. The formation of these stripe-shaped films was explained by the schematic illustration in Figure 8C by the authors.

#### 3.3.4. NCD Composites with other Materials

Except for Ni/NCD, Al/NCD and Fe/NCD composites, NCD composites with other metals have shown excellent tribological properties, such as Mg [125,126], Ti [127,128,129,130], Cu [131,132], etc. These NCD composites, prepared by sintering, can significantly improve the tribological properties of drills, tools, workpieces, etc. Especially for Ti metal, owing to its lightweight, high hardness and biocompatibility, it is a perfect material in the field of medical application [133,134]. The preparation of Ti/NCD composites will further improve its performance and expand its application range, for example, the lubrication of artificial joints for medical surgery [129,130,131,132]. Due to the biocompatibility of both NCD and Ti [34,135,136], Chen et al. [60] demonstrated that a novel two-dimensional (2D) diamond nanosheet coating on Ti alloys synergistically enhances friction and wear performance combined with natural silk fibroin.

## 4. Simulation of NCD in Tribology

In order to better investigate the mechanisms and influencing factors of friction and wear in NCD materials, the introduction of simulation research is as essential as experimental evidence. MD, a common simulation method in material science, was widely employed to simulate the tribological behavior in experiments and to offer a profound understanding of the mechanisms and influencing factors by combining its findings with experimental results. More importantly, the MD can also anticipate experiments that have not been started so that efficient experiments can be better carried out [137]. Hu et al. [138] used MD LAMMPS to investigate the frictional properties of two hard nanoparticles (diamond and SiO_2_) and reveal the mechanisms governing their anti-wear and friction-reducing behavior. Research showed that ball-bearing, polishing and support effects existed at low velocity and low load. At low velocity and high load, the rolling effect does not exist because the NCD particles were squashed. At high velocity and low load, the support effect existed for a certain duration. At high velocity and high load, the transfer layer is formed for a short time. Mirzaamiri et al. [81] investigated the tribological properties and influencing factors of NCD particles in aqueous suspensions through a series of experiments and MD simulations. The results showed that the introduction of NCD particles significantly improved the naturally poor lubrication ability of water. The best performance was characterized by reductions of 70% and 88% in friction and wear, respectively. The enhancement of the tribological performance was mainly attributed to the ball-bearing effect of the NCD particles. It can be seen that the integration of work using experimental and computational models is important for exploring the mechanisms of NCD in tribology.

## 5. Conclusions

This paper reviews the application of NCD in the field of tribology. The current applications of NCD can be divided into three areas according to different lubricating types, namely lubricant additives, lubricating films and lubricating composite materials.

1. For NCD additives, tribological performances were enhanced through the polishing effect of the lubricant containing NCD powders on the friction surface, as well as the ball-bearing effect during the rubbing, leading to a wide range of applications for NCD powders in the field of lubrication additives. NCD powder additives in oil, water or even a body fluid exhibited outstanding tribological properties. The concentration of NCD powder additives is a critical factor that influences tribological performance. Hence, it is necessary to probe the optimized concentration and improve the dispersity of NCD additives.
2. For NCD lubricating films, the COF of single-layer NCD films was significantly reduced. The films can be deposited on a variety of substrates to expand the application of the NCD lubricating films. The poor adhesion between the NCD lubricating film and the substrate is a subject that needs to be further studied.
3. For NCD composite materials, its outstanding properties of high hardness, small size, chemical stability and high thermal conductivity makes NCD considered a candidate for reinforcing materials. The incorporation of NCD into the metal coating increased functionality and improved the tribological properties. NCD can be sintered with other materials to produce end mills, drills, wear parts, etc. Although most studies of NCD composites have shown improvements in tribological properties, the current NCD composites do not perform as well in terms of tribology as expected.

## 6. Outlook

NCD has an excellent performance in tribology, which has led to great interest in its tribological applications. At present, NCD is mainly studied in tribology in the forms of lubricating additives, lubricating films and composite coatings. Although the research has achieved obvious results in various aspects, it is mostly in the development stage and has only a small amount of practical applications. NCD powders need further improvement on dispersion, uniformity and compatibility of solutes in terms of lubricating additives. For lubricating NCD films, there are challenges in the improvement of the adhesive strength between film and substrate, as well as the lubricating effect. The friction reduction and anti-wear performance of the NCD composite coating and the bonding with the matrix still are key points to be addressed. With the development of technology, the performance requirements in tribology are becoming more and more demanding for NCD lubricating materials. New areas of friction, such as highly loaded dynamic seal faces for deep-sea machinery, safe and reliable body fluid lubricants, as well as wear-resistant and non-delaminating artificial joints, have led to a deeper and more extensive study of NCD. The integration of experiment and simulation methods will enable us to further investigate the friction mechanisms and develop new applications of NCD in the field of tribology. Furthermore, the preparation methods are being optimized, and the performance is being improved, expanding the scope of commercialization of NCD in the field of tribology.

## Figures and Tables

**Table 3:** Summary of tribological performance for NCD lubricating composites.

Lubricating System	Tribological Test and Performance					Refs.
	Method	Environment	Counterpart	COF (Original)	Wear (Original)	
electroplating m-Ni/NCD on AISI-1045 steel	ball-on-disk	ambient conditions, 3 N, 65 mm/s	AISI 52100 steel	\	1.3 × 10^−4^ mm^3^/N·m (2.5 × 10^−4^ mm^3^/N·m)	[116]
electroplating n-Ni/NCD on AISI-1045 steel				\	6 × 10^−5^ mm^3^/N·m (3 × 10^−5^ mm^3^/N·m)	[116]
Sintering Ni/25wt% NCD on stainless steel	pin-on-disc	ambient conditions, 1 N, 30 mm/s	WC-Co	\	3.4 × 10^−6^ mm^3^/N·m (4.3 × 10^−4^ mm^3^/N·m)	[119]
cold spraying Al/2wt% NCD on AISI 1020 steel	ball-on disk	ambient conditions, 1 N, 10 rpm/min	Al_2_O_3_	~ 0.74 ± 0.021	\	[123]
cold spraying- heat treating Al/2wt% NCD on AISI 1020 steel				~ 1.15 ± 0.013	\	[123]
hot-press sintering Fe/1wt% NCD	ball-on-disc	ambient conditions, 30 N, 3.3 Hz	GCr15 steel	~0.5 (~0.8)	~0.12 mm^3^ (~0.38 mm^3^)	[124]
spark plasma sintering Ti/2 wt% NCD	wear ring electronic tester	50–200 N	GCr15 steel	\	1.9% (0.4%)	[127]
spark plasma sintering Ti/2.5 wt% NCD	friction-abrasion tester	3 N, 320 rpm	Si_3_N_4_	0.503–0.674 (0.654)	6.349–8.016 mg (12.101 mg)	[128]

## Data Availability

Not applicable.
